# Liposome Consolidated with Cyclodextrin Provides Prolonged Drug Retention Resulting in Increased Drug Bioavailability in Brain

**DOI:** 10.3390/ijms21124408

**Published:** 2020-06-21

**Authors:** En-Yi Lin, Yu-Shuan Chen, Yuan-Sheng Li, Syuan-Rong Chen, Chia-Hung Lee, Mao-Hsuan Huang, Hong-Meng Chuang, Horng-Jyh Harn, Hsueh-Hui Yang, Shinn-Zong Lin, Dar-Fu Tai, Tzyy-Wen Chiou

**Affiliations:** 1 Department of Life Science, National Dong Hwa University, No. 1, Sec. 2, Da Hsueh Rd., Shou-Feng, Hualien 974301, Taiwan; dora871010@gmail.com (E.-Y.L.); s9123849@gmail.com (Y.-S.L.); sharonk0607@gmail.com (S.-R.C.); chlee016@gms.ndhu.edu.tw (C.-H.L.); 2 Department of Chemistry, National Dong Hwa University, No. 1, Sec. 2, Da Hsueh Rd., Shou-Feng, Hualien 974301, Taiwan; 3 Bioinnovation Center, Buddhist Tzu Chi Medical Foundation, Hualien 970, Taiwan; yushuanchenxie@gmail.com (Y.-S.C.); spleo0825@gmail.com (M.-H.H.); kavin273@gmail.com (H.-M.C.); arthewduke@gmail.com (H.-J.H.); shinnzong@yahoo.com.tw (S.-Z.L.); 4 Department of Medical Research, Hualien Tzu Chi Hospital, Buddhist Tzu Chi Medical Foundation, Hualien 970, Taiwan; hhyang@tzuchi.com.tw; 5 Department of Stem Cell Applied Technology, Gwo Xi Stem Cell Applied Technology, Hsinchu 30261, Taiwan; 6 Laboratory of Translational Medicine Office, Development Center for Biotechnology, Taipei 115, Taiwan; 7 Department of Pathology, Hualien Tzu Chi Hospital, Tzu Chi University, Buddhist Tzu Chi Medical Foundation, Hualien 970, Taiwan; 8 Department of Neurosurgery, Hualien Tzu Chi Hospital, Buddhist Tzu Chi Medical Foundation, Hualien 970, Taiwan

**Keywords:** glioblastoma multiforme, butylidenephthalide, cyclodextrin, liposome, stability, bioavailability

## Abstract

Although butylidenephthalide (BP) is an efficient anticancer drug, its poor bioavailability renders it ineffective for treating drug-resistant brain tumors. However, this problem is overcome through the use of noninvasive delivery systems, including intranasal administration. Herein, the bioavailability, drug stability, and encapsulation efficiency (EE, up to 95%) of BP were improved by using cyclodextrin-encapsulated BP in liposomal formulations (CDD1). The physical properties and EE of the CDD1 system were investigated via dynamic light scattering, transmission electron microscopy, UV–Vis spectroscopy, and nuclear magnetic resonance spectroscopy. The cytotoxicity was examined via MTT assay, and the cellular uptake was observed using fluorescence microscopy. The CDD1 system persisted for over 8 h in tumor cells, which was a considerable improvement in the retention of the BP-containing cyclodextrin or the BP-containing liposomes, thereby indicating a higher BP content in CDD1. Nanoscale CDD1 formulations were administered intranasally to nude mice that had been intracranially implanted with temozolomide-resistant glioblastoma multiforme cells, resulting in increased median survival time. Liquid chromatography–mass spectrometry revealed that drug biodistribution via intranasal delivery increased the accumulation of BP 10-fold compared to oral delivery methods. Therefore, BP/cyclodextrin/liposomal formulations have potential clinical applications for treating drug-resistant brain tumors.

## 1. Introduction

Glioblastoma multiforme (GBM), which is classified as a Grade IV tumor by the World Health Organization, is an aggressive and highly progressive disease that is often fatal. In general, GBM has a mean survival rate of approximately 8 to 15 months after diagnosis [1]. In a population-based study, only 2.2% of the patients survived more for than two years [2], and the 5-year overall survival rate is generally below 10% [3]. Although efforts have been devoted to treating GBM patients, the average survival rate remains around 14.6 months, despite using multimodal treatment strategies such as surgery, radiotherapy, and combined chemotherapy using TMZ [1]. Surgical resection is a standard treatment approach after the diagnosis of GBM; however, the residual tumor is hard to remove, and normal tissue is generally damaged during the surgical process. Combination therapy with radiotherapy/chemotherapy is a treatment option, but the severity of the accompanying side effects and the persistence of chemical-resistant cells can hamper progress. Despite being a standard treatment, TMZ is inefficient, particularly for patients with O6-methylguanine–DNA methyltransferase (MGMT) expression [4,5,6]. Because these issues contribute to the recurrence of GBM [1,7,8], there is an urgent need to develop new therapies to combat these problems.

Butylidenephthalide (BP; molecular weight ~188.22) is a small, hydrophobic molecule that is found in the chloroform extract of *Angelica sinensis*, which is also called “danggui” in traditional Chinese medicine. BP is an effective anticancer agent that induces apoptosis, inhibits telomerase, and retards the metastasis of tumor cells [9,10,11,12]. BP initiates GBM apoptosis through p53-dependent and -independent pathways via a cell-cycle blockade in the G0/G1 phase [9]. Additionally, BP has been shown to induce apoptosis via the protein kinase C (PKC)-mediated activation of Nur77 by localizing the cell nucleus; once activation is complete, BP is translocated to the cytosol. The cytosolic Nur77 protein binds to the antiapoptosis protein Bcl-2, thereby causing conformational changes in the mitochondria that trigger the release of cytochrome c and activates caspase 3 to induce cell apoptosis [10]. Furthermore, BP inhibits telomerase activation, which accelerates cell senescence and apoptosis, thereby leading to tumor toxicity [11]. BP’s primary role is to enhance the methylation of the MGMT promoter, thereby decreasing the expression of MGMT, ultimately leading to cell toxicity in TMZ-resistant GBM [13].

A problem usually encountered in cancer treatment is the blood–brain barrier (BBB), which limits the efficiency of the administered chemotherapy [14]. BBB is typically bypassed by employing a noninvasive strategy involving an intranasal delivery system that takes advantage of the nose-to-brain transport system [15]. Here, intranasal administration has been used to treat brain tumors via the delivery of siRNA, antisense oligonucleotides (e.g., GRN163), vascular stomatitis virus, neural stem and progenitor cells, 5-fluorouracil, and methotrexate in preclinical trials. Intranasal administration is often used in clinical trials to deliver drugs as the monoterpene perillyl alcohol analog [16]. Unfortunately, the hydrophobic nature of BP limits nasal drug delivery owing to its low fluidity. BP is unstable in the presence of oxygen, which results in a decrease in cytotoxicity. To broaden the range of potential applications for disease treatment and to prevent its oxidation, a new drug-carrier system for BP was designed in this study.

Cyclodextrin, which is composed of sugar molecules bound together to form a ring structure with a truncated cone shape, is widely used as a drug carrier. In particular, β-cyclodextrins composed of seven-membered sugar ring molecules such as (2-hydroxypropyl)-β-cyclodextrin are often used as intranasal enhancers [17]. The inner cavity of a cyclodextrin molecule is hydrophobic, whereas the outer surface is hydrophilic [18,19]. This hydrophobic cavity provides a space to load hydrophobic drugs, whereas the hydrophilic surface is beneficial for drug solubility [18,20]. Cyclodextrin improves the stability of hydrophobic drugs by minimizing their exposure to oxygen [21]. However, drug delivery only using this system does not display the hallmarks of a controlled release system. Therefore, the development of formulations, which include other carriers that can facilitate controlled drug release and accumulation in brain tissues, is essential.

Liposomes, which have a lipid bilayer structure similar to the cell membrane, have proved suitable for carrying hydrophobic molecules within their lipid bilayer while facilitating hydrophilic drugs in their cavities [22,23]. Liposomes are used as carriers for doxorubicin in anticancer therapy, which has been approved by the FDA and marketed as Doxil [24]. One advantage of using liposomes as carriers is that they prolong the circulation time of drugs [25]. However, the amount of hydrophobic drug that can be loaded is limited by the space of the lipid bilayer. This problem can be overcome by taking advantage of cyclodextrin’s hydrophilic and hydrophobic properties [26,27]. Cyclodextrin-carrying BP forms hydrophilic inclusion complexes that can occupy the liposome’s cavity, thereby increasing the drug-loading capacity. Accordingly, a BP/cyclodextrin/liposome system could be envisioned for expanding the drug-loading capacity and improving its controlled release.

In this study, a BP/cyclodextrin/liposome system was developed and characterized in terms of its physical properties, i.e., size, structural appearance, and encapsulation efficiency (EE). The cytotoxicity of the newly developed approach was examined using the 3-(4,5-dimethylthiazol-2-yl)-2,5-diphenyltetrazolium bromide (MTT) assay. The cellular uptake of the BP/cyclodextrin/liposome formulations was observed via fluorescence microscopy to establish drug retention. Finally, the therapeutic efficiency of the formulations in the treatment of brain cancer was examined in mice bearing drug-resistant glioblastoma multiforme. The amount of BP in brain tissue samples was further investigated using liquid chromatography–mass spectrometry (LC–MS) to analyze drug biodistribution.

## 2. Results

### 2.1. Characteristics of Drug Formulations

The drug encapsulation efficiencies for each BP/(2-hydroxypropyl)-β-cyclodextrin (CD)/liposome formulation (Table 1) were calculated using Equation (1) (see Section 4.6); they are given along with their physical properties in Table 2. The particle size for each formulation ranged from 211.2 ± 0.60 to 360.9 ± 23.06 nm for the percentages of the encapsulated drug from 18% to 25%. The polydispersity index (PDI) values for all formulations were below 1, meaning that the formulations were homogenous suspensions in the respective solution.

### 2.2. Appearance of the Liposomes

**Figure 1:**
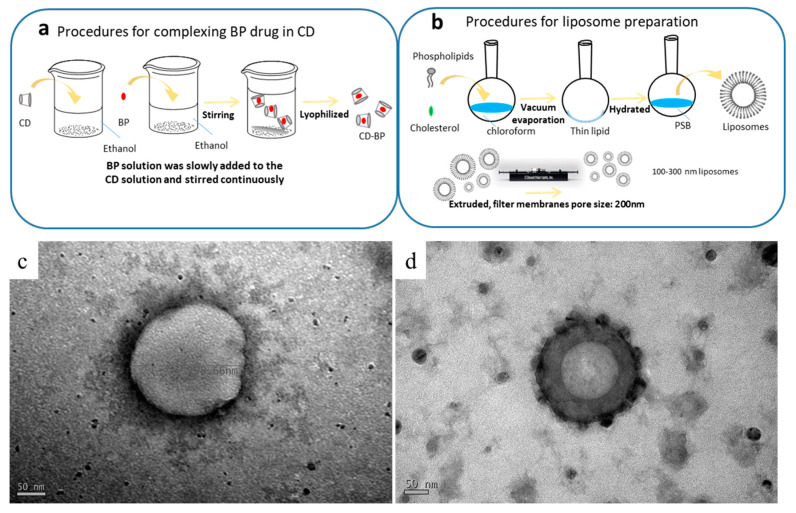
Schematic of the complexation of (**a**) the BP drug in the CD (abbreviated as BP/CD) and (**b**) the liposomal (abbreviated as LipoD) systems. TEM characterization of the formulations’ structures for (**c**) LipoD and (**d**) the CDD1 formulations comprising LipoD and BP/CD. Scale bars for (**c**,**d**) are 50 nm.

The structures of the formulations were observed using TEM. The spherical structures seen in Figure 1 had diameters ranging from 200 to 250 nm. The CDD1 formulation (Figure 1d) showed a more compact bilayer structure than the structure associated with the “liposome only” sample (LipoD, Figure 1c). Moreover, black particles were deposited on or integrated into the outer surface of the CDD1 sphere, indicating that the (2-hydroxypropyl)-β-cyclodextrin (CD) molecule may have been either deposited on the liposomes or displayed synergistic effects with the liposomes [28] that resulted in the stabilization of the liposome-encapsulated BP. Both the cavity and the internal space of the bilayer lipid membrane in the liposome are represented in gray in the figure below and are indicative of the encapsulation of the BP drug in this biological transport vehicle.

### 2.3. Characterization of Formulations and the Encapsulation Efficiency

**Figure 2:**
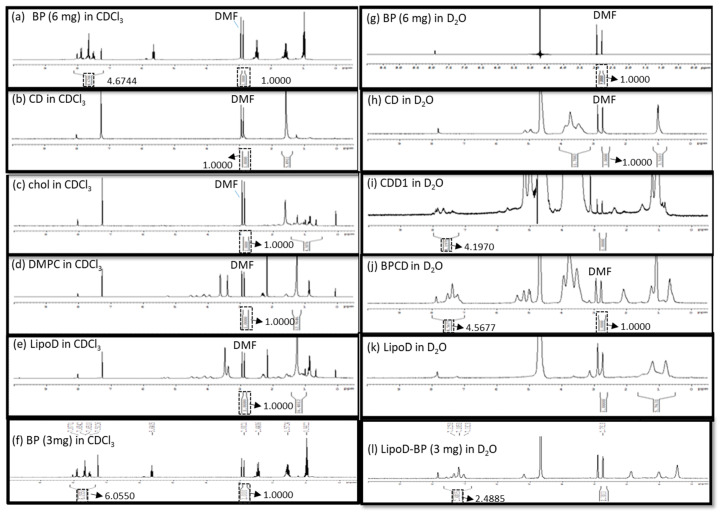
Characterization of (**a**) 6-mg BP in CDCl_3_, (**b**) the CD molecule in CDCl_3_, (**c**) cholesterol in CDCl_3_, (**d**) DMPC in CDCl_3_, (**e**) LipoD comprising DMPC and cholesterol in CDCl_3_, (**f**) 3-mg BP in CDCl_3_, (**g**) 6-mg BP in D_2_O, (**h**) the CD molecule in D_2_O, (**i**) CDD1 comprising LipoD and BP (6 mg)/CD in D_2_O, (**j**) BP (6 mg)/CD complex in D_2_O, (**k**) LipoD comprising DMPC and cholesterol in D_2_O, and (**l**) LipoD/BP (3 mg) in D_2_O formulations via NMR spectroscopy. The quantification of BP’s encapsulation efficiency was conducted using DMF as the internal standard.

The formation of the inclusion complex of BP/CD was confirmed via nuclear magnetic resonance (NMR), Fourier transform infrared (FTIR) spectroscopy, and visual inspection. As pure BP is a yellow liquid, a failed inclusion complex showed distinct phases comprising the yellow BP liquid and a white lyophilized powder CD after lyophilization. By comparison, successful complexation was evidenced by yellow lyophilized powder. Moreover, the NMR results showed that the BP drug was only soluble in a hydrophobic solvent such as chloroform-d (CDCl_3_), which, in turn, caused the aromatic hydrogens of BP to undergo chemical shifts at approximately 7–8 ppm (Figure 2a); however, this phenomenon was not observed in the hydrophilic solvent deuterium oxide (D_2_O) (Figure 2g). The CD molecule was soluble in D_2_O and exhibited chemical shifts between 3.4 and 3.8 ppm that corresponded to the H2–H6 CH protons of CD (Figure 2h) [29], which were not observed when the analysis was conducted in CDCl_3_ (Figure 2b). When BP was mixed with the CD molecule, the chemical shifts noted at 7–8 ppm were attributed to BP’s aromatic groups, which were observed in D_2_O. Moreover, a polymer-like complex was formed, as evidenced by the broad peak noted at 7–8 ppm in the spectrum obtained for the BP/CD formulation relative to the spectrum obtained for pure BP (Figure 2j). These results demonstrated that the BP drug was soluble in a hydrophilic solvent in the presence of the CD molecule. The formation of a BP/CD inclusion complex was confirmed via FTIR spectroscopy. The –OH stretching band and bending vibrations, as well as the –HOH stretching bands of the pure CD molecule (Figure S1), which afforded peaks at 3412, 1035, and 1645 cm^−1^, respectively, were shifted or broadened after the formation of the BP/CD inclusion complex (Figure S1). These results provided proof that the BP/CD inclusion complex had been formed.

The CD/*Angelica sinensis* complex was previously characterized via NMR to confirm the occurrence of interactions between these molecules [18]. As BP is an active component in *Angelica sinensis*, we conducted NMR analysis to measure the EE in the formulations. Here, *N*,*N*-dimethyl formamide (DMF) served as the internal standard for quantifying the BP content in the respective formulations. Briefly, BP and BP/CD were dissolved in CDCl_3_ and D_2_O, respectively, at a concentration of 6 mg/500 μL each, and the resulting integration areas observed at 7–8 ppm in the spectra were compared. The integrals of the aromatic signals were 4.6744 (Figure 2a) and 4.5677 (Figure 2j) in CDCl_3_ and D_2_O, respectively, and were obtained by normalizing the integration area of DMF (2.8–2.9 ppm) to 1. The ratio of the encapsulated BP in CD was 97.6% in a chemical preparation of 1:2.5 (*w*/*w*).

The chemical interaction between BP’s EE and the cyclodextrin/liposome (CDD1) system was also assessed via NMR. Here, no aromatic signals were observed at 7–8 ppm for LipoD (Figure 2e), cholesterol (Figure 2c), and DMPC (Figure 2d). The CDD1 system exhibited signals at 7–8 ppm in D_2_O (Figure 2i) that were not observed in the CD (Figure 2h) or the LipoD (Figure 2k) spectra in D_2_O. By comparing the integral area of BP’s aromatic group in CDCl_3_ (4.6744) and the CDD1 (4.1970) system, the calculated ratio of the encapsulated BP in CDD1 was 89.7%, thereby providing further proof that BP could be formulated with CDD1 or CD molecules and was soluble in a hydrophilic environment.

A comparison of the EE of BP in the liposomes was also conducted via NMR analysis. Here BP (3 mg) was dissolved in CDCl_3_, and the liposomes encapsulated with 3 mg of BP (LipoD/BP) were dissolved in D_2_O. The integral areas of the aromatic signals were 6.0550 and 2.4855 for BP in CDCl_3_ (Figure 2f) and LipoD/BP in D_2_O (Figure 2l), respectively. The ratio of the encapsulated BP to the liposome was 0.411 and was calculated using the corresponding NMR integrals (2.4855/6.0550). Accordingly, the actual weight of BP in the liposome was 1.233 mg (3 mg × 0.411), indicating that 1 mg of the liposome contained 0.411 mg of BP. In contrast, the ratio of the encapsulated BP increased to 0.898 when the integration area was 4.1970 (Figure 2a)/4.6744 (Figure 2l) of CD in the liposomal system. A BP value of 5.382 mg (6 mg × 0.898) was evidence of the molecule’s successful introduction into the CDD1 system, i.e., every 1 mg of the liposome contained 1.794 mg of BP. Therefore, a 4.47-fold increase in the amount of BP present in the system was observed when the CD molecule was introduced into the liposomal system.

The interaction of BP/CD in the liposome and the role of CD in stabilizing the liposome’s structure were investigated via NMR spectroscopy. Previous studies have shown that the long hydrocarbon chains of the liposome were embedded in the hydrophobic region of CD [28], which was strikingly similar to the results obtained in the current study. Accordingly, signals corresponding to the liposomes were observed at 1.5–2.3 ppm for the acyl-CH_2_ and 3.0–4.3 ppm in the CDD1 composition (Figure 2i). In contrast, such resonances were not observed in the LipoD system in D_2_O (Figure 2k), indicating that either the CD molecule had been deposited on the liposome or the long hydrophobic chains of the liposome had been incorporated into the hydrophobic region of CD, which, in turn, stabilized the encapsulated BP. We used nanoparticle tracking analysis (NTA) to determine that the particle size distribution was helpful for evaluating the liposome (LipoD) (Figure S2a), liposome BP (LipoD/BP) (Figure S2), and the CDD1 (Figure S2) formulation stabilization. The CDD1 formulation possessed a single peak after 1, 6, and 14 days of storage at 4 °C. However, LipoD and LipoD/BP exhibited multiple peaks after 1, 6, and 14 days of storage at 4 °C. From these results, it was concluded that CD stabilized the liposome’s structure.

### 2.4. Cytotoxicity in Drug-Resistant Glioblastoma Multiforme Cells

**Figure 3:**
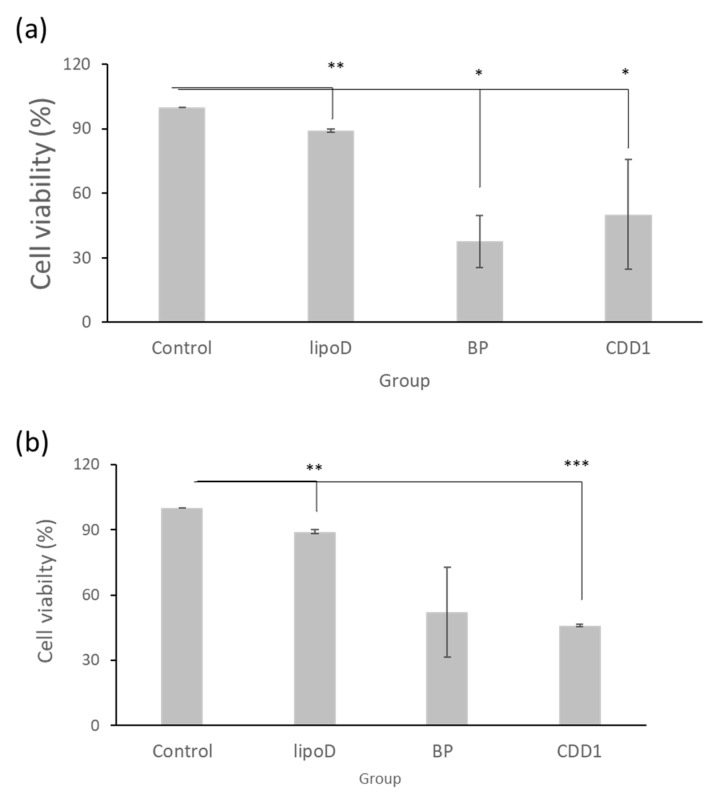
Cell viability of GBM22–TMZ in the (**a**) freshly prepared formulations and (**b**) formulations stored at 4 °C for two weeks. Control indicates the medium group, lipoD indicates cholesterol/DMPC, BP represents the BP drug formulated in DMSO, and CDD1 indicates 25% BP encapsulated in CD and packed into cholesterol/DMPC. *** represents *p* < 0.001, ** represents *p* < 0.01, and * represents *p* < 0.05.

Our drug delivery system’s cytotoxicity was examined via treatment with GBM22–TMZ, a drug-resistant glioblastoma multiforme cell line. Here, with the medium sample as the control and liposome D (LipoD) as the vehicle, BP was formulated in dimethyl sulfoxide (DMSO) and the CDD1 system. We noted that the viability of the GBM22–TMZ cells was greater than 90% in the LipoD only group (no drug) and was comparable with the results observed for the control group (Figure 3a). The viability of the BP- and CDD1-treated cells was 37% and 46.5%, respectively. The cytotoxicity triggered by the release of BP from the CDD1 system was slightly lower than that associated with the free BP group (Figure 3a).

The storage stability of the formulations was tested using cytotoxicity measurements via GBM22–TMZ. The formulations were stored at 4 °C for two weeks prior to cell treatments before MTT assays were performed. The LipoD vehicle was not toxic to GBM22–TMZ cells when compared with the control group. Interestingly, when the cells were treated with BP formulated in DMSO, the viability of the GBM22–TMZ cells was 52% (Figure 3b), representing lower toxicity than that associated with the freshly prepared BP formulation (Figure 3a). In contrast, treatment with CDD1 resulted in 46% cell viability, which was similar to the results obtained for the freshly prepared CDD1 formulation (Figure 3b). The results demonstrated that the liposome coating the CD system protected the drug and provided functional stability.

### 2.5. Dynamic Cellular Uptake of the Formulations

**Figure 4:**
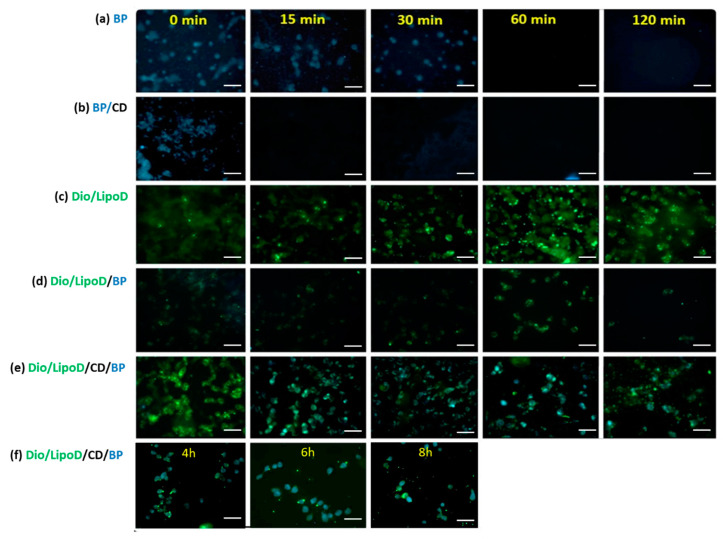
Fluorescence microscopy of the cellular uptake in GBM8401 after treatment with the respective formulations. (**a**) Uptake of BP (blue) formulated in DMSO from 0 to 120 min. (**b**) Uptake of BP (blue)/CD from 0 to 120 min. (**c**) Uptake of Dio/liposome (green), abbreviated as Dio/LipoD, from 0 to 120 min. (**d**) Uptake of Dio/liposome (green)/BP (blue), abbreviated as Dio/LipoD/BP, from 0 to 120 min. (**e**) Uptake of Dio/liposome (green)/CD/BP (blue), abbreviated as Dio/LipoD/CD/BP, from 0 to 120 min and (**f**) continuously from 4 to 8 h. All the scale bars are 50 μm.

The uptake and release of BP or the BP formulations by the GBM8401 cells were monitored by first treating the cells with BP or the formulated BP and making observations over a specific timeframe (Figure 4). As BP exhibits autofluorescence upon excitation at 355 nm and emission at 435 nm [30], we monitored the cellular BP content via fluorescence microscopy. The BP formulated in CD (i.e., BP/CD) entered the GBM8401 cells rapidly, as confirmed by the blue color observed in the cells at 0 min (Figure 4b). After 15 min, considerably less BP was present in the cells with the BP/CD formulation, suggesting that this system could not sustain delivery (Figure 4b). In contrast, observations of the free BP drug dissolved in DMSO bore results until the 30-min time point (Figure 4a). This may be due to the interaction between the hydrophobic drug and the hydrophilic phase of the cytoplasm, which served to prolong the drug occupancy in the cells. The liposome vehicle labeled with Dio dye (green color) also penetrated the cells very quickly, from as early as 0 min (Figure 4c), as did the liposome formulation system (Figure 4c–e). The liposome formulation system sustained the release of BP in the GBM8401 cells (Figure 4d–f). In the “liposome only” group, the carried BP molecules (noted as cyan color when derived from the blue and green colors) produced results until the 120-min time point, which indicated that the liposome system could protect BP for a longer period of time in the cell (Figure 4d). When the CD and liposome systems were combined, the carried BP molecules were observed in the cells at least 8 h (Figure 4e–f). These findings supported the theory that a cyclodextrin-consolidated liposome system protected the carried drug molecules in the cellular environment, thereby facilitating the sustained release of the therapeutic agent.

### 2.6. In Vivo Animal Survival Rate

**Figure 5:**
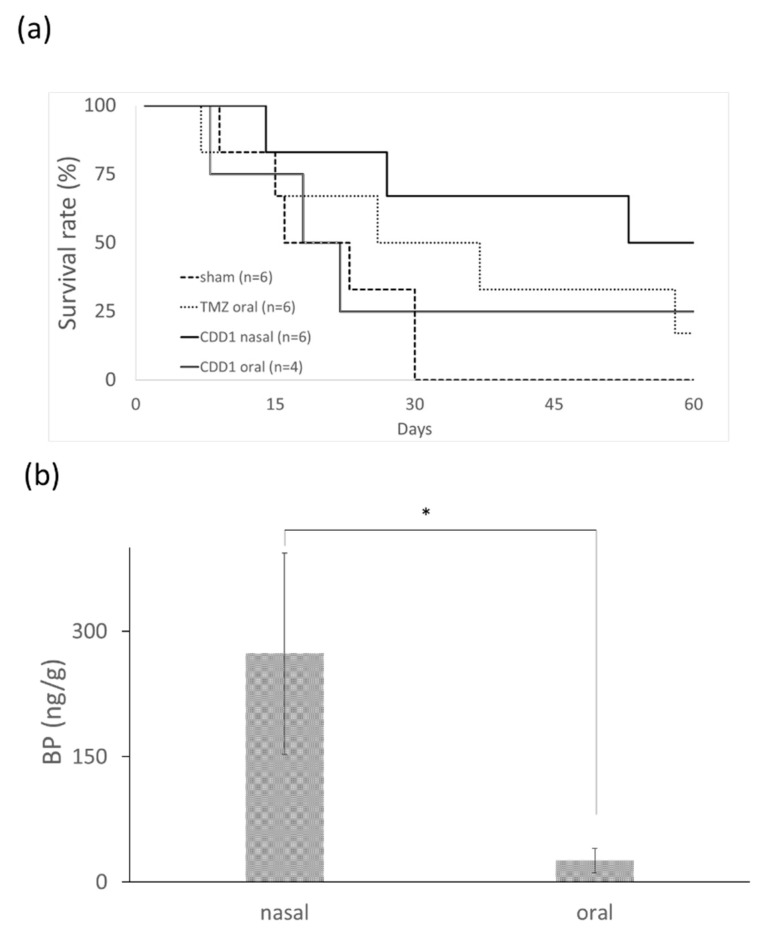
Survival rate and BP quantity in brain. (**a**) Survival rate of mice bearing drug-resistant glioblastoma multiforme after the oral administration of TMZ (66 mg/kg/day for 5 consecutive days), after the oral or nasal administration of CDD1 (50 mg/kg/day for 14 consecutive days), and without treatment (sham). (**b**) Quantification of BP levels in brain tissue samples (*n* = 8) 30 min after the drug was administered with CDD1 (the BP amount was 1 mg) via the nose or mouth. * represents *p* < 0.00005.

The efficiency of each formulation for the treatment of brain cancer was evaluated using nude mice bearing drug-resistant glioblastoma multiforme. New drugs for the treatment of TMZ-resistant brain tumors were screened using mouse models harboring a TMZ-resistant brain tumor. The dose and treatment period for the mice were determined from previously reported human trials [31] by employing the conversion formula for the animal dose = human dose × (37/3) [32]. The differences in the bioavailability and the metabolic rate of each drug led us to conclude that a TMZ dosage of 66 mg/kg/day for five consecutive days was appropriate [31]. The median survival time after therapy was compared for four groups: the control group without any drug treatment, the group undergoing oral administration of TMZ, the group undergoing oral administration of CDD1, and the group undergoing nasal administration of CDD1. The data depicted in Figure 5a shows that the nasally administered CDD1 group exhibited the longest median survival time of 60 days when compared with the control group (22 days), the TMZ-treated group (36 days), and the orally administered CDD1 group (21 days).

### 2.7. In Vivo BP Levels in Brain Tissue

A prolonged survival rate was observed in mice bearing TMZ-resistant GBM subjected to nasally administered CDD1. The distribution of BP in the brain tissue was measured via LC–MS to determine the quantity of BP present 30 min after nasal or oral administration. The concentration of BP in the brain tissue was 273.19 ± 120.47 and 26.68 ± 14.87 ng/g (brain tissue weight) when administered nasally or orally, respectively. The quantity of BP obtained from the brain tissue samples was approximately 10-fold higher than the quantity obtained from the nasally administered tissue samples. These findings demonstrate that BP formulated with the CD/liposome system could be delivered in high concentrations to the brain via nasal administration.

## 3. Discussion

In nude mice animal models, brain tumors were treated using a subcutaneously administered cocktail containing BP, DBTRG–05MG cells (dose: 70–800 mg/kg), polymer wafers with DBTRG–05MG cells (dose: 37.5–125 mg/kg), and intracranial polymer wafers in spontaneous brain tumors (dose: approximately 37.5–187.5 mg/kg). Athymic mice underwent treatment via intravenous (IV) administration of DBTRG–05MG cells (dose: 100 mg/kg) [9,30,33]. In this study, we successfully developed a formulation that could noninvasively deliver a hydrophobic anticancer drug via an intranasal route using a dose of 50 mg/kg, which was less than the dosage used in subcutaneous IV administration. The anticancer drug BP is a viable candidate for the treatment of TMZ-resistant brain cancer. The median survival time observed after treatment with the intranasally administered CDD1 formulation was 60 days, which was much longer than the results observed in the control or the orally administered CDD1 groups (Figure 5a). As previously reported, delivery via the intranasal route facilitated 1- to 1.5-fold and 1.9- to 3.1-fold higher concentrations of the therapeutic agent than the results noted for IV and oral administration, respectively [34]. As a high dose of BP in the brain tissue samples was achieved via intranasally administered CDD1 (Figure 5b), as evidenced by the 10-fold higher concentration of the therapeutic agent compared to the orally administered counterpart, it can be reasonably concluded that the addition of a nasal enhancer such as CD will significantly improve the effectiveness of our formulation [35].

The optimized formulation should have been screened out at the beginning of our study because the IC_50_ results obtained for BP in the treatment of GBM were around 75 μg/mL, a value that indicated lower cytotoxicity than that of marketable anticancer drugs that had nanogram-scale dosages. However, as our study’s objective was to improve the drug payload, a variety of customized formulations were developed and analyzed via UV–Vis spectroscopy. Here, we noted that CDD1 showed the highest drug payload of all the formulations tested, thereby making it a candidate for subsequent studies in which the drug-loading capacity was compared in the “liposome only” or liposome/CD formulations via NMR spectroscopy.

There are slight differences in the EE results obtained via UV–Vis and NMR spectroscopic analysis, which can be attributed to the signal overlap between the liposome and BP at 310 nm, which resulted in higher EE values in the UV–Vis analysis relative to the results from NMR spectroscopy. Although the liposome exhibited absorption at 310 nm, the composition ratio of the liposome in the CDD1, CDD2, and CDD3 formulations was the same. UV–Vis spectroscopy was used to determine which formation possessed the highest drug payload. However, because bias existed only if the formulations did not contain the same composition ratio as the liposome, NMR spectroscopy was applied to evaluate the EE of BP. The NMR analysis of CDD1 was performed thrice (data not shown), and the average EE of BP in the CDD1 was 85.1%. Monitoring of the batch bias for each formulation via NMR spectroscopy, with DMF as the internal control, was undertaken in CDCl_3_ using equal aliquots of each formulation.

A liposomal system was previously used to encapsulate BP with an encapsulation percentage of 4.8% [30]. In our study, a higher weight percentage of BP (4.47-fold higher) was achieved using the CDD1 formulation when compared with the “liposome only” formulation. These phenomena were consistent with the results of previous studies, in which the EE was improved by formulating the drug into CD prior to the encapsulation of the drug/CD complex in the liposome [27].

In previous reports, the cellular uptake of CD/*Angelica sinensis* extract complex was observed within 1 h [18]. In our research, we noted the persistent release of BP when the liposome or CD/liposome was applied. Hagiwara et al. reported that the release of doxorubicin from the liposome/CD complex was slower than the results observed in the “liposome only” group [36]. Our results may be attributed to several factors, including the prolonged pharmaceutic drug control release facilitated by the liposome and the enhanced structural stability of the liposome, which was brought about by the drug/CD complex via deposition on the liposome’s surface. Evidence to support this hypothesis was obtained by TEM analysis.

The stability of our system was also exemplified by the cytotoxicity results obtained (Figure 3b). When BP was formulated in the liposome/CD system and stored for two weeks, the noted cytotoxicity of the formulation was similar to that of the freshly prepared formulations. In contrast, BP formulated in DMSO exhibited decreased cytotoxicity in the tested cancer cells (Figure 3), indicating that CDD1 played a role in the stability of BP. Moreover, NTA analysis revealed a single peak for the CDD1 formulation after 1, 6, and 14 days of storage, whereas liposome D and liposome BP exhibited multiple peaks after 1, 6, and 14 days of storage.

## 4. Materials and Methods

### 4.1. Materials

1,2-Dimyristoyl-sn-glycero-3-phosphocholine (DMPC, 14:0 PC, AV-850345P, Avanti Polar Lipids, Inc. Alabaster, Alabama), cholesterol (C3292, Sigma–Aldrich, St. Louis, MO, USA), (2-hydroxypropyl)-β-cyclodextrin (CD, 332607, Sigma–Aldrich, St. Louis, MO, USA), butylidenephthalide (BP, Alfa Aesar, Göteborg, Sweden), and 3,3′-dihexadecyloxacarbocyanine perchlorate (Dio, Molecular Probe, Inc., Junction, USA) were commercially obtained.

### 4.2. Encapsulation of BP

The procedure for preparing the inclusion body of BP/CD is summarized in Figure 1a. Here, the encapsulation of BP into CD was achieved by first dissolving CD (2.5 g) in 60 mL of 95% ethanol. In a separate container, BP (1 g) was dissolved in 15 mL of 95% ethanol, and the resulting solution was slowly added to the CD solution with continuous stirring overnight at 4 °C. The thoroughly mixed BP/CD solution was then frozen and lyophilized overnight using a UNISS FDM-2 at −85 °C and 10 mTorr to obtain the BP/CD complex in its lyophilized form.

### 4.3. Preparation of Liposomes

The procedure for preparing the liposomes is summarized in Figure 1b. As shown in Table 1, the DMPC:cholesterol ratio was 11:3 (g/g). The two components were dissolved in chloroform, and the final concentration of the mixture was 1 mg/mL. The flask was then connected to a Büchi R205 rotary evaporator (Flawil, Switzerland) equipped with a 40 °C water bath. The solvent was removed under vacuum to give a homogeneous lipid film that coated the flask’s wall, which was then kept under continuous vacuum for another 2 h to remove all residual chloroform. The lipid film was then hydrated with PBS by sonication in a water bath at 24 °C, which is the DMPC transition temperature. After 1.5 h, the hydrated lipid film formed a homogeneous dispersion of liposomes that were subsequently extruded at room temperature using a mini-extruder (Whatman, Inc., Clifton, NJ, USA) and 0.2-μm polycarbonate filters. The extruded liposome was stored overnight at 4 °C for future experiments.

### 4.4. Preparation of BP-Containing Liposomes

Liposomes containing BP were prepared using the respective weights given in Table 1. Here, BP or the above-mentioned BP/CD complex was added to the liposomes to obtain the liposome BP (abbreviated as LipoD/BP) or CDD1–CDD3 by ultrasonication in a water bath at 24 °C for 10 min. The BP-containing liposomes and the BP/CD complex were stored at 4 °C for future experiments.

### 4.5. Particle Size and Size Distribution Coefficient Analysis

Dynamic light scattering (DLS) analysis [37] was conducted to determine the size of the particles dispersed in the diluted suspension. Here, the formulations were prepared with a BP concentration of 200 μg/mL in deionized (DI) water before measurements were performed using a Zetasizer Nano ZS (Nano-ZS, ZEN3600, Malvern Panalytical, UK). The average particle size and PDI of the freshly prepared formulations were measured (*n* = 3, mean ± standard deviation (SD)).

### 4.6. EE Analysis by UV–Vis Spectroscopy

The EE of the drug was determined to establish the drug content in the respective formulations. Purification via high-speed centrifugation was performed before measurements were obtained. Briefly, the BP-containing liposomes were dispersed in PBS to remove any residual BP in the supernatant via high-speed centrifugation at 13,400 rpm. The purification process was performed four times, after which UV–Vis spectroscopy was conducted on the supernatant to confirm the absence of BP. In our case, the BP-containing liposomes were measured via UV–Vis spectroscopy at 310 nm. All experiments were conducted in triplicate. EE was defined as the ratio of the actual and original amounts of the liposomal formulation containing BP, and was determined as follows:

EE = (W_t_/W_i_) × 100% (1)

where W_t_ is the actual amount of the active drug in the formulation, which was detected by UV–Vis spectroscopy at 310 nm, and W_i_ is the amount of the active drug added during the preparation process.

### 4.7. EE Analysis via NMR Spectroscopy

The solubility of BP in the formulation was determined using NMR analysis. All solutions containing BP, i.e., the pure BP, BP/CD, and BP-containing liposome formulations, were dissolved in D_2_O or CDCl_3_ to examine their solubility in each deuterated solvent. The same amount of DMF was added to each analyzed solution as an internal control, and the integration area of DMF was set at 1. The weights of BP, CD, and liposome D were 6, 15, and 3 mg, respectively. The same amount of each component was weighed for the BP/CD and CDD1 formulations, i.e., a total of 21 and 24 mg, respectively. For comparison, the EE value of BP in the “liposomes only” sample was also determined using 3 mg each of BP and liposome D. The samples were dissolved in 500-μL D_2_O or CDCl_3_ for NMR spectroscopy.

### 4.8. TEM Analysis

The BP-containing liposomes were diluted 500-fold with pure water before an aliquot of 10 μL of the resulting solution was applied to carbon-coated 400 mesh copper grids (Electron Microscopy Sciences, Hatfield, PA, USA) for 60 s. Any excess solution was removed using a Kimwipe^TM^ [38]. The grids were negatively stained by incubation with a 10-μL drop of 1.5% phosphotungstic acid for another 60 s before the excess solution was removed using a Kimwipe^TM^. The grids of the BP-containing liposomes were stored in a desiccator for future analysis. All samples were analyzed at 120 kV using a JEOL JEM-1200CX II.

### 4.9. MTT Assay for Cytotoxicity

MTT colorimetric assay (Sigma–Aldrich, St. Louis, MO, USA) was used to determine cell survival. The GBM22–TMZ cells, a kind gift from Sarkaria JN at The Mayo Clinic, were seeded in 96-well plates (Falcon, Arizona, USA) with a cell density of 5 × 10^3^ cells in 100 μL per well. After cell seeding overnight, the BP formulations were prepared at a concentration of 75 μg/mL and added to the cells for 48 h. BP encapsulation in CDD1 was confirmed via UV–Vis absorption at 310 nm before cell treatment. The storage stability of the formulations was examined using the same method, and the applied formulations were stored at 4 °C for two weeks prior to treatments. The control group was treated with the same amount of medium. After a 48-h incubation period, 100 μL of the MTT solution was added per well at a concentration of 500 μL/mL. The MTT solution was incubated with the cells for 1.5 h before 50 μL of DMSO was added per well to dissolve the crystals formed in the viable cells. The absorbance of the purple solution was measured at 595 nm after a 10-min incubation period on a shaker. All MTT experiments were performed in duplicate with a total of eight runs for each sample.

### 4.10. Drug Uptake in Tumor Cells

The Dio/liposomes (Dio/LipoD) were prepared using the method reported by Chekhun et al. [39]. Briefly, the liposomes and the Dio were prepared at concentrations of 0.7785 and 1 mM, respectively, in chloroform. The Dio/liposomes were mixed well before the solvent was removed using a rotary evaporator. The resulting Dio/liposomes were resuspended in PBS solution and subjected to sonication in a water bath for 10 min. The Dio/liposomes served as the raw phospholipid materials for the construction of the Dio/CDD1 system. The cellular uptake of the drug of interest, i.e., BP, BP/CD, Dio/liposome/BP (Dio/LipoD/BP), and the Dio/CDD1 (Dio/LipoD/CD/BP) systems, was assessed using GBM 8401 cell lines. After diluting the drug in the medium to obtain a BP concentration of 2 mg/mL, the labeled formulations were incubated with GBM8401 cells to observe the dynamic processes of cellular uptake via fluorescence microscopy (Boston Microscopes, Inc., Wilmington, USA).

### 4.11. Malignant Drug-Resistant Brain Tumor Animal Models and Treatment of Nude Mice

Nude mice (BALB/cAnN.Cg-Foxn1nu/CrlNarl) were purchased from the National Laboratory Animal Center (NLAC). All animal experiments were conducted according to the animal protection laws established by the National Dong Hwa University Animal Management Commission under approval IACUC No. 103002. For this animal model, the mice (7–9-week-old males) were anesthetized with 4% chloral hydrate (10 μL/g) and placed in a stereotaxic apparatus. The cranium was exposed by creating a sagittal incision in the skin, and a small burr hole was carefully made 1.5 mm laterally and 2 mm caudally to the bregma. Then, 1 × 10^6^ cells were delivered using a 27G Hamilton syringe (Hamilton, Reno, NV, USA) at a depth of 2.5 mm through the parenchyma [40]. Six microliters of TMZ-resistant brain tumor cell lines (GBM22–TMZ) that had been suspended in PBS were manually injected at a rate of 0.5 μL/min. Five days after the surgical procedure, the animals were randomly grouped as follows: (a) untreated, (b) orally administered with TMZ at a concentration of 66 mg/kg/day for 5 consecutive days [33], (c) orally administered with CDD1 and BP concentration of 50 mg/kg/day for 14 consecutive days, and (d) intranasally administered with CDD1 and BP concentration of 50 mg/kg/day for 14 consecutive days. The drug was delivered through the nostrils using a 20-µL-long tip in a dropwise manner until a total volume of 20 µL was completely delivered under anesthesia. Each drop was retained for at least 5 s or until it was no longer visible in the nostril, and the drug was delivered through a different nostril each day. Body weight and survival time were recorded daily.

### 4.12. Biodistribution

#### 4.12.1. Drug Administration

The mice were administered CDD1 with a total BP amount of 1 mg via nasal or oral routes; the delivery volume was 20 or 200 μL (*n* = 8 for each group). The mice were sacrificed 30 min after the administration of the drug, and their brains were collected and weighed before storage at −80 °C.

#### 4.12.2. Sample Preparation for LC–MS Analysis

BP was extracted by first roughly chopping tissue samples with a pair of scissors and placing the pieces in 2 mL of chloroform. The chopped tissue samples were homogenized using a motorized pellet pestle, followed by ultrasonication in a water bath for 5 min at room temperature. The supernatant was harvested from the homogenized tissue by centrifugation at 2000 rpm for 10 min before being dried using flowing nitrogen gas to obtain dry BP pellets. The BP residue in the tissue pellets was concentrated by two additional runs of ultrasonication and nitrogen gas drying. The extracted BP was harvested in the same tube and reconstituted using acetonitrile at a concentration of 1 mg tissue/μL. The samples were stored at −80 °C for further analysis.

#### 4.12.3. LC–MS Analysis

A UPLC system (Vanquish UPLC Systems, Thermo–Fisher) was used to establish the drug content of the samples. Chromatographic separation was performed using a 1.9-μm Hypersil-Gold column (2.1 mm × 100 mm, Waltham, USA). The column temperature was 35 °C, the flow rate was between 0.3 and 0.45 μL/min, and the injection volume was 20 μL. The mobile phases A and B were composed of water and acetonitrile, respectively, with 0.1% formic acid in both phases. In the UPLC gradient profile, the mobile phase B was 5% (*v*/*v*) at 0 min, which was then linearly increased to 100% from 0 to 3.5 min before returning to 5% from 3.5 to 8 min. For the mobile phase A, the gradient at 95% (*v*/*v*) at 0 min decreased to 0% from 0 to 3.5 min before returning to 95% from 3.5 to 8 min. MS detection was performed on a Q Exactive^TM^ plus LC–MS/MS system (Thermo–Fisher) equipped with an electrospray ionization source (ESI) operating in the positive ionization mode. High-resolution data acquisition was performed for a single reaction in the parallel reaction monitoring mode using an *m/z* of 189.0903/128.0618 for BP. The optimized instrument parameters for monitoring the analytes via MS were as follows: S lens voltage, 50 V; ion spray voltage, 3800 V; source temperature, 350 °C; and Sheath/Aux/Sweep gas, 38/5/1 psi. We used blank brain tissue samples (i.e., untreated brain tissue) as the gold-standard matrix. Standardized curves were plotted for the integrated area against various BP concentrations that were dissolved in the blank brain tissue matrix to mimic the actual reaction conditions. Credible results were obtained by conducting the same experimental procedures with the standard curves to obtain validated interday and intraday assay results.

### 4.13. Statistical Analysis

Data were analyzed using the Student’s t-test. Statistical significance was noted when *p* ≤ 0.05. All data are presented as the mean ± SD.

## 5. Conclusions

In summary, a new strategy using a CDD1 formulation was developed to deliver BP to the brain. Here, the drug-loading capacity was improved by the incorporation of a CD molecule. Sustained release and increased drug stability were facilitated by a liposome system. The nanoformulated hydrophobic drug CDD1, which was approximately 360 nm in size, could be administered intranasally to achieve successful delivery to the brain. Our results indicate that the CDD1 system is a potential candidate for brain tumor therapy applications, particularly for drug-resistant GBM.

## Figures and Tables

**Table 1:** Composition of the formulations.

Formulation	DMPC mg (%)	CL mg (%)	BP mg (%)	CD mg (%)
LipoD	55 (78.57%)	15 (21.43%)	0 (0%)	0 (0%)
CDD1	55 (9.82%)	15 (2.68%)	140 (25%)	350 (62%)
CDD2	55 (17.46%)	15 (4.76%)	70 (22%)	175 (55.56%)
CDD3	55 (28.57%)	15 (7.79%)	35 (18.18%)	87.5 (45.45%)

Abbreviations: DMPC: 1,2-dimyristoyl-sn-glycero-3-phosphocholine; CL: cholesterol; BP: butylidenephthalide; CD: (2-hydroxypropyl)-β-cyclodextrin.

**Table 2:** Physical properties of the formulations and the efficiency of drug encapsulation.

Formulation (BP%)	Z-Average (nm)	PDI	%EE (±SD)
LipoD (0%)	211.2 ± 0.60	0.08 ± 0.03	0
CDD3 (18%)	253.0 ± 6.05	0.33 ± 0.01	58.2 ± 2.3
CDD2 (22%)	331.4 ± 4.74	0.47 ± 0.06	89.9 ± 1.0
CDD1 (25%)	360.9 ± 23.06	0.53 ± 0.07	98.4 ± 3.7

Abbreviation: PDI: polydispersity index; BP%: initiative weight percentage of BP; %EE: encapsulation efficiency (%); LipoD: comprising DMPC and cholesterol; CDD: BP represents the (2-hydroxypropyl)-β-cyclodextrin (CD)/LipoD formulation.

## Supplementary Materials

Supplementary materials can be found at https://www.mdpi.com/1422-0067/21/12/4408/s1. Figure S1. Characterization of the structure of (a) (2-hydroxypropyl)-β-cyclodextrin (CD) and (b) Butylidenephthalide (BP)/CD inclusion complex via Fourier transform infrared spectroscopy. Figure S2. Evaluation of the stability of (a) LipoD, comprising DMPC and cholesterol, in deionized (DI) water; (b) LipoD-BP, comprising BP and liposome, in DI water; (c) Cyclodextrin-encapsulated BP into liposomal formulations (CDD1) in DI water storage at 4 °C for 1, 6, and 14 days via NTA.

## Abbreviations

EE	Encapsulation efficiency
ESI	Electrospray ionization
MS	Mass spectrometry
NLAC	National Laboratory Animal Center
NMR	Nuclear magnetic resonance
NTA	Nanoparticle tracking analysis
SD	Standard deviation
TEM	Transmission electron microscopy
TERT	Telomerase reverse transcriptase

## References

[1] Anjum K., Shagufta B.I., Abbas S.Q., Patel S., Khan I., Shah S.A.A., Akhter N., Hassan S.S.U. (2017). Current status and future therapeutic perspectives of glioblastoma multiforme (GBM) therapy: A review. Biomed. Pharmacother..

[2] Scott J.N., Rewcastle N.B., Brasher P.M.A., Fulton D., MacKinnon J.A., Hamilton M., Cairncross J.G., Forsyth P. (1999). Which glioblastoma multiforme patient will become a long-term survivor? A population-based study. Ann. Neurol..

[3] Scott J.N., Rewcastle N.B., Brasher P.M., Fulton D., Hagen N.A., MacKinnon J.A., Sutherland G., Cairncross J.G., Forsyth P. (1998). Long-term glioblastoma multiforme survivors: A population-based study. Can. J. Neurol. Sci. (J. Canadien Sci. Neurol.).

[4] Janzer R.C., Raff M.C. (1987). Astrocytes induce blood-brain barrier properties in endothelial cells. Nature.

[5] Sun Y.H., Zhang Y.Z., Wang Z.C., Sun M.Z., Zhao D.H. (2004). [Relationship between the expression of O6-methylguanine-DNA methyltransferase in glioma and the survival time of patients]. Ai Zheng.

[6] Hegi M.E., Diserens A.C., Gorlia T., Hamou M.F., de Tribolet N., Weller M., Kros J.M., Hainfellner J.A., Mason W., Mariani L. (2005). MGMT gene silencing and benefit from temozolomide in glioblastoma. N. Engl. J. Med..

[7] Bondy M.L., Scheurer M.E., Malmer B., Barnholtz-Sloan J.S., Davis F.G., Il’yasova D., Kruchko C., McCarthy B.J., Rajaraman P., Schwartzbaum J.A. (2008). Brain tumor epidemiology: Consensus from the Brain Tumor Epidemiology Consortium. Cancer.

[8] Roy S., Lahiri D., Maji T., Biswas J. (2015). Recurrent Glioblastoma: Where we stand. South Asian J. Cancer.

[9] Tsai N.M., Chen Y.L., Lee C.C., Lin P.C., Cheng Y.L., Chang W.L., Lin S.Z., Harn H.J. (2006). The natural compound n-butylidenephthalide derived from Angelica sinensis inhibits malignant brain tumor growth in vitro and in vivo. J. Neurochem..

[10] Lin P.C., Chen Y.L., Chiu S.C., Yu Y.L., Chen S.P., Chien M.H., Chen K.Y., Chang W.L., Lin S.Z., Chiou T.W. (2008). Orphan nuclear receptor, Nurr-77 was a possible target gene of butylidenephthalide chemotherapy on glioblastoma multiform brain tumor. J. Neurochem..

[11] Lin P.C., Lin S.Z., Chen Y.L., Chang J.S., Ho L.I., Liu P.Y., Chang L.F., Harn Y.C., Chen S.P., Sun L.Y. (2011). Butylidenephthalide suppresses human telomerase reverse transcriptase (TERT) in human glioblastomas. Ann. Surg. Oncol..

[12] Huang M.H., Lin S.Z., Lin P.C., Chiou T.W., Harn Y.W., Ho L.I., Chan T.M., Chou C.W., Chuang C.H., Su H.L. (2014). Brain tumor senescence might be mediated by downregulation of S-phase kinase-associated protein 2 via butylidenephthalide leading to decreased cell viability. Tumour Biol. J. Int. Soc. Oncodevelopment. Biol. Med..

[13] Yen S.Y., Harn H.J., Lin S.Z., Chen S.R., Lin P.C., Syu F.J., Hsieh D.K., Huang M.H., Chiou T.W. (2012). (Z)-butylidenephthalide Restores Temozolomide Sensitivity to Temozolomide-resistant Malignant Glioma Cells by Downregulating Expression of the DNA Repair Enzyme MGMT. Eur. J. Cancer.

[14] Abbott N.J., Ronnback L., Hansson E. (2006). Astrocyte-endothelial interactions at the blood-brain barrier. Nat. Rev. Neurosci..

[15] Dhuria S.V., Hanson L.R., Frey W.H. (2010). Intranasal delivery to the central nervous system: Mechanisms and experimental considerations. J. Pharm. Sci..

[16] Van Woensel M., Wauthoz N., Rosiere R., Mathieu V., Kiss R., Lefranc F., Steelant B., Dilissen E., Van Gool S.W., Mathivet T. (2016). Development of siRNA-loaded chitosan nanoparticles targeting Galectin-1 for the treatment of glioblastoma multiforme via intranasal administration. J. Control Release.

[17] Merkus F.W., Verhoef J.C., Marttin E., Romeijn S.G., van der Kuy P.H., Hermens W.A., Schipper N.G. (1999). Cyclodextrins in nasal drug delivery. Adv. Drug Deliv. Rev..

[18] Hsu C.M., Tsai F.J., Tsai Y. (2014). Inhibitory effect of Angelica sinensis extract in the presence of 2-hydroxypropyl-beta-cyclodextrin. Carbohydr. Polym..

[19] Tiwari G., Tiwari R., Rai A.K. (2010). Cyclodextrins in delivery systems: Applications. J. Pharm. Bioallied Sci..

[20] Arima H., Yunomae K., Miyake K., Irie T., Hirayama F., Uekama K. (2001). Comparative studies of the enhancing effects of cyclodextrins on the solubility and oral bioavailability of tacrolimus in rats. J. Pharm. Sci..

[21] Arima H., Miyaji T., Irie T., Hirayama F., Uekama K. (1998). Enhancing effect of hydroxypropyl-beta-cyclodextrin on cutaneous penetration and activation of ethyl 4-biphenylyl acetate in hairless mouse skin. Eur. J. Pharm. Sci..

[22] Akbarzadeh A., Rezaei-Sadabady R., Davaran S., Joo S.W., Zarghami N., Hanifehpour Y., Samiei M., Kouhi M., Nejati-Koshki K. (2013). Liposome: Classification, preparation, and applications. Nanoscale Res. Lett..

[23] Beck Z., Matyas G.R., Alving C.R. (2015). Detection of liposomal cholesterol and monophosphoryl lipid A by QS-21 saponin and Limulus polyphemus amebocyte lysate. Biochim. Biophys. Acta.

[24] Barenholz Y. (2012). Doxil(R)--the first FDA-approved nano-drug: Lessons learned. J. Control Release.

[25] Ashby L.S., Smith K.A., Stea B. (2016). Gliadel wafer implantation combined with standard radiotherapy and concurrent followed by adjuvant temozolomide for treatment of newly diagnosed high-grade glioma: A systematic literature review. World J. Surg. Oncol..

[26] Zhang L., Zhang Q., Wang X., Zhang W., Lin C., Chen F., Yang X., Pan W. (2015). Drug-in-cyclodextrin-in-liposomes: A novel drug delivery system for flurbiprofen. Int. J. Pharm..

[27] Gharib R., Greige-Gerges H., Fourmentin S., Charcosset C., Auezova L. (2015). Liposomes incorporating cyclodextrin-drug inclusion complexes: Current state of knowledge. Carbohydr. Polym..

[28] Ikeda A., Iwata N., Hino S., Mae T., Tsuchiya Y., Sugikawa K., Hirao T., Haino T., Ohara K., Yamaguchi K. (2015). Liposome collapse resulting from an allosteric interaction between 2,6-dimethyl-β-cyclodextrins and lipids. RSC Adv..

[29] Zhao R., Tan T., Sandström C. (2011). NMR studies on puerarin and its interaction with beta-cyclodextrin. J. Biol. Phys..

[30] Lin Y.-L., Chang K.-F., Huang X.-F., Hung C.-L., Chen S.-C., Chao W.-R., Liao K.-W., Tsai N.-M. (2015). Liposomal n-butylidenephthalide protects the drug from oxidation and enhances its antitumor effects in glioblastoma multiforme. Int. J. Nanomed..

[31] Villano J.L., Seery T.E., Bressler L.R. (2009). Temozolomide in malignant gliomas: Current use and future targets. Cancer Chemother. Pharmacol..

[32] Pandy V. (2020). A Simple Method for Animal Dose Calculation in Preclinical Research. EC Pharmacol. Toxicol..

[33] Harn H.J., Lin S.Z., Lin P.C., Liu C.Y., Liu P.Y., Chang L.F., Yen S.Y., Hsieh D.K., Liu F.C., Tai D.F. (2011). Local interstitial delivery of z-butylidenephthalide by polymer wafers against malignant human gliomas. Neuro Oncol..

[34] Zhao Y., Yue P., Tao T., Chen Q.H. (2007). Drug brain distribution following intranasal administration of Huperzine A in situ gel in rats. Acta Pharmacol. Sinica.

[35] Rassu G., Soddu E., Cossu M., Brundu A., Cerri G., Marchetti N., Ferraro L., Regan R.F., Giunchedi P., Gavini E. (2015). Solid microparticles based on chitosan or methyl-β-cyclodextrin: A first formulative approach to increase the nose-to-brain transport of deferoxamine mesylate. J. Controll. Release Off. J. Controll. Release Soc..

[36] Arima H., Hagiwara Y., Hirayama F., Uekama K. (2006). Enhancement of antitumor effect of doxorubicin by its complexation with gamma-cyclodextrin in pegylated liposomes. J. Drug Target..

[37] Naiim M., Boualem A., Ferre C., Jabloun M., Jalocha A., Ravier P. (2015). Multiangle dynamic light scattering for the improvement of multimodal particle size distribution measurements. Soft Matter.

[38] Owen J., Stride E. (2015). Technique for the Characterization of Phospholipid Microbubbles Coatings by Transmission Electron Microscopy. Ultrasound Med. Biol..

[39] Yefimova S.L., Kurilchenko I.Y., Tkacheva T.N., Rozhkov V.A., Sorokin A.V., Lukianova N.Y., Bezdenezhnykh N.A., Malyukin Y.V., Chekhun V.F. (2012). Comparative study of dye-loaded liposome accumulation in sensitive and resistant human breast cancer cells. Exp. Oncol..

[40] Baumann B.C., Benci J.L., Santoiemma P.P., Chandrasekaran S., Hollander A.B., Kao G.D., Dorsey J.F. (2012). An Integrated Method for Reproducible and Accurate Image-Guided Stereotactic Cranial Irradiation of Brain Tumors Using the Small Animal Radiation Research Platform. Transl. Oncol..

