# Supplementary material for: Liposome Consolidated with Cyclodextrin Provides Prolonged Drug Retention Resulting in Increased Drug Bioavailability in Brain
Source: Int J Mol Sci. 2020 Jun 21;21(12):4408. doi: 10.3390/ijms21124408 (PMC7352271; doi:10.3390/ijms21124408)
Supplement: Supplementary file 1 [file ijms-21-04408-s001.pdf]

International Journal of
Molecular Sciences

MDPI

Materials and Methods

Inclusion complex analysis by Fourier transform infrared spectrometry (FTIR)

FTIR analysis was performed using the KBr pellet method. Each liposomal sample was lyophilized. KBr powder (Thermo, Waltham, MA, USA) was heated in an oven at 100 °C for 24 h to eliminate residual moisture and further used as a blank. Liposome samples were mixed with KBr at a mass ratio of approximately 1:99 and compressed into a thin section, and the percent transmittance was determined in the range of 500–4000 cm^{-1} using an FTIR spectrophotometer (Alpha FT-IR Spectrometer, Bruker, USA).

Formulation stability via nanoparticle tracking analysis (NTA)

The size distribution of liposomes was determined via NTA using an NS 300 Nanosight™ (Nanosight, UK) instrument to evaluate liposome stability. Liposomes were resuspended in deionized (DI) water and analyzed. All default settings were applied with the viscosity set to 0.90–0.95 cP and the temperature set to 22 °C–25 °C. A single measurement comprised three 60 s videos, each sample was evaluated five times at camera level 13, and data acquisition and processing were performed using the NTA3.0 software (Nanosight). Only recordings with over 1000 valid tracks/vesicles were included in the analysis.

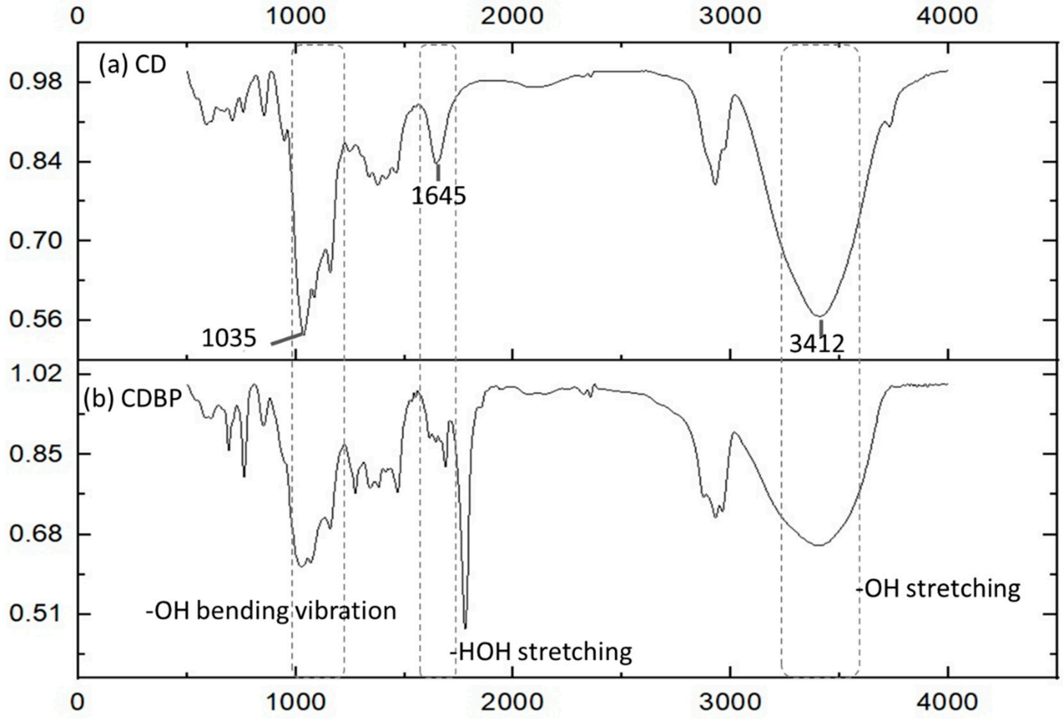

Figure S1. Characterization of the structure of (**a**) (2-hydroxypropyl)-β-cyclodextrin (CD) and (**b**) Butylidenephthalide (BP)/CD inclusion complex via Fourier transform infrared spectroscopy.

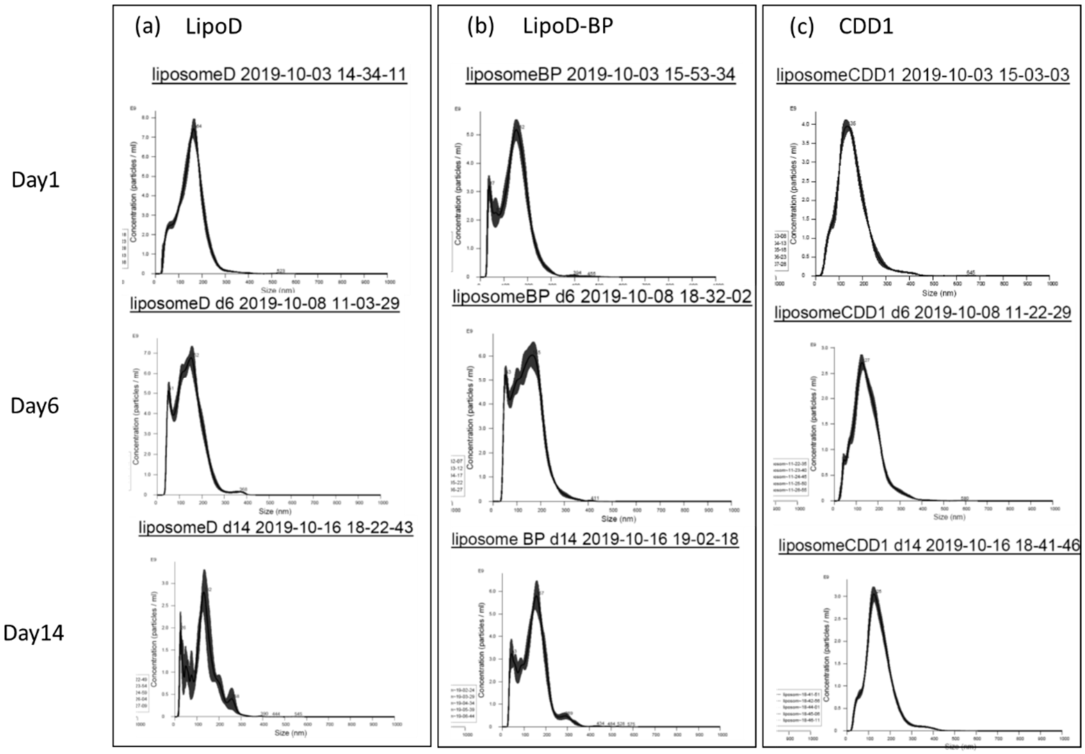

Figure S2. Evaluation of the stability of (**a**) LipoD, comprising DMPC and cholesterol, in deionized (DI) water; (**b**) LipoD-BP, comprising BP and liposome, in DI water; (**c**) Cyclodextrin-encapsulated BP into liposomal formulations (CDD1) in DI water storage at 4 °C for 1, 6, and 14 days via NTA.
